# An Approach Based on the Protected Object for Dam-Break Flood Risk Management Exemplified at the Zipingpu Reservoir

**DOI:** 10.3390/ijerph16193786

**Published:** 2019-10-08

**Authors:** Congxiang Fan, Ruidong An, Jia Li, Kefeng Li, Yun Deng, Yong Li

**Affiliations:** State Key Laboratory of Hydraulics and Mountain River Engineering, Sichuan University, 24 South Section 1, Ring Road No. 1, Chengdu 610065, China; 2017323060024@stu.scu.edu.cn (C.F.); kefengli@scu.edu.cn (K.L.); dengyun@scu.edu.cn (Y.D.); liyong@scu.edu.cn (Y.L.)

**Keywords:** dam-break flood, risk management, numerical simulation, classification method, Zipingpu Reservoir

## Abstract

Dam-break flooding is a potential hazard for reservoirs that poses a considerable threat to human lives and property in downstream areas. Assessing the dam-break flood risk of the Zipingpu Reservoir in Chengdu, Sichuan Province, China, is critically important because this reservoir is located on the Longmen Shan fault, which experiences high seismic activity. In this paper, we develop an approach based on the protected object for dam-break flood risk management. First, we perform a numerical simulation of dam-break flooding in four possible dam break scenarios. Next, the flood areas are divided into 71 analysis units based on the administrative division. Based on the numerical simulation results and the socio-economic demographic data affected by a flood, the importance and risk level of each analysis unit is confirmed, and the flood risk map is established according to the classification results. Finally, multi-level flood risk management countermeasures are proposed according to the results of the unit classification shown in the map.

## 1. Introduction

The Zipingpu Reservoir is situated in the upper reach of the Minjiang River in Sichuan Province, China, approximately 60 km east of Chengdu, the capital of Sichuan Province. The Zipingpu Reservoir is a large water control project with comprehensive benefits, such as irrigation and water supply, power generation, and flood control, in addition to being a Dujiangyan irrigation district water source adjustment project. The Zipingpu Reservoir is located on the Longmen Shan fault, which experiences high seismic activity. Several strong earthquakes have occurred in this area in recent years, such as the 2008 Wenchuan earthquake (Mw 8.0) and the 2013 Lushan earthquake (Mw 7.0) [1] The Wenchuan earthquake, with an epicenter approximately 17 km away from the Zipingpu Dam, caused cracks in the dam panel, powerhouse, and other buildings, causing the walls of the hydropower station to collapse and undergo local subsidence. As a result, the entire power plant unit was shut down. The impact intensity at the dam site was IX-X, which raised the awareness toward the potential risk. Although there is minimal probability of a dam break, its occurrence would be disastrous for people’s lives and property and for indigenous ecosystems of the downstream city of Chengdu [2], which is the technology, trade, and financial center of southwest China. Therefore, many studies have focused on determining how to effectively reduce the occurrence of dam failure and reduce the losses caused by a dam break. At present, research on reservoir reinforcement measures is becoming mature, and engineering measures, such as engineering reinforcement for the dam, have been increasingly adopted. However, in the construction of a flood control system, a flood disaster cannot be easily controlled by relying on engineering measures, and the extreme natural conditions that can cause a dam breaks cannot be effectively resisted. Therefore, non-engineering measures, such as the evolution process of dam break floods and dam-break flood risk management plans, must also be studied.

Procedures for assessing flood risk and flood evolution process analyses have always been used to study flood scales and disaster mitigation measures for dam-break floods [3]. Many national institutions attach great importance to flood risk management and have set up large-scale flood risk map projects for risk prediction management [4]. Existing research on flood risk management mainly focuses on the mechanism and evolution of dam-break floods [5,6], assessments of direct and indirect losses of life and property in downstream areas affected by a flood [7,8], and the establishment of risk and emergency management measures [9,10]. Previous research has focused on improving hydraulic calculations and post-disaster loss evaluation methods. The shallow water equations have been widely used in the study of dam-break flood and has been proved to be robust and effective. In recent studies, the numerical integration of the equations of motion is based on shock capturing schemes. Castro-Orgaz and Chanson used MUSCL-Hancock finite volume method and the discontinuous Galerkin finite-element method to get two accurate solutions of the viscous dam break wave propagating over a dry-bed [11]. Delis et al. compared the cell-centered and node-centered unstructured finite volume (CCFV, NCFV) discretizations for shallow water free surface flows and found that the quality of the mesh primarily affect the convergence performance of the CCFV, but NCFV has a consistent performance on the different grid types used [12]. Cannata et al. reported a dam-break flood model based on a contravariant integral form of the Shallow Water Equations, which used a high-order WENO reconstruction procedure [13]. Breaking wave propagation in the surf zone is simulated by integrating the nonlinear shallow water equations with a high-order shock-capturing scheme [14]. The mapping of flood risks is an essential component of flood risk management; thus, Meyer et al. developed a GIS-based multicriteria flood risk assessment and mapping approach including economic, environmental, and social risks [15]; Neal et al. analyzed the spatial dependence between rivers for flood risk mapping [16]; and Alfonso et al. proposed a flood risk map based on the concept of the value of information (VOI) to explore the use of uncertain information, which assists in spatial planning decision-making [17]. Risk management is a systematic project led by the government [18]. However, previous studies have largely ignored that the implementation of flood risk management is led by an administrative unit; thus, flood risk management measures cannot be effectively implemented, and the allocation of government resources is not reasonable. From the perspective of service in urban management, the subject of flood protection and management is the administrative unit. In this regard, this paper proposes a flood risk management method based on an administrative unit combined with the classification of flood hazards and unit importance. The specific steps of the proposed method are as follows:

1. Analyze the possibility of occurrence of a dam-break flood disaster in the specific area and simulate the spatial propagation of the dam-break flood. In this case, the potential hazard caused by the dam-break flood can be more clearly reflected [19].
2. The risk of a flood disaster is the result of the comprehensive effect of the flood hazard and the environmental vulnerability [20]. This paper generalizes these two properties into the flood hazard and unit importance. The hazard and importance level of each analysis unit is classified based on the numerical simulation results and socio-economic demographic data of the flood-affected areas.
3. Formulate countermeasures for flood control management in the downstream areas of the reservoir, considering the flood risk of the dam break according to the different hazards and importance levels.

## 2. Numerical Simulation of Dam-Break Flood Evolution in the Zipingpu Reservoir Downstream Area 

### 2.1. Numerical Simulation Model 

The simulation is based on a two-dimensional hydrodynamic model considering the average water depth [21,22]. The numerical simulation is performed on MIKE software.

(1) Equation of continuity

(1) $$\frac{\partial h}{\partial t}+\frac{\partial h\bar{u}}{\partial x}+\frac{\partial h\bar{v}}{\partial y}=0$$

(2) Momentum equation

(2) $$\frac{\partial h\bar{u}}{\partial t}+\frac{\partial h{\bar{u}}^{2}}{\partial x}+\frac{\partial h\bar{uv}}{\partial y}=\frac{\partial }{\partial x}\left(2h\upsilon_{t}\frac{\partial \bar{u}}{\partial x}\right)+\frac{\partial }{\partial x}\left(h\upsilon_{t}\left(\frac{\partial \bar{u}}{\partial y}+\frac{\partial \bar{v}}{\partial x}\right)\right)+f\bar{v}h-gh\frac{\partial \xi }{\partial x}-\frac{h}{\rho }\frac{\partial P_{a}}{\partial x}-\frac{\tau_{bx}}{\rho }$$

(3) $$\frac{\partial h\bar{v}}{\partial t}+\frac{\partial h{\bar{v}}^{2}}{\partial y}+\frac{\partial h\bar{uv}}{\partial x}=\frac{\partial }{\partial x}\left(2h\upsilon_{t}\frac{\partial \bar{v}}{\partial y}\right)+\frac{\partial }{\partial x}\left(h\upsilon_{t}\left(\frac{\partial \bar{u}}{\partial y}+\frac{\partial \bar{v}}{\partial x}\right)\right)+f\bar{u}h-gh\frac{\partial \xi }{\partial y}-\frac{h}{\rho }\frac{\partial P_{a}}{\partial y}-\frac{\tau_{by}}{\rho }$$

where *t = time; h = total depth, h =ξ + d*; *ξ = water surface elevation; d = static total depth*; g = acceleration of gravity; *ρ* = water density; $\upsilon_{t}$ = eddy viscosity coefficient; *f =* Coriolis force parameter; *τ_bx_* and *τ_by_* = frictional stress in the x and y directions, respectively; *u* and *v* = depth average velocity in the x and y directions, respectively; and $h\bar{u}=\int_{-d}^{\eta }udz$ $h\bar{v}=\int_{-d}^{\eta }vdz$.

(3) Eddy viscosity coefficient

The eddy viscosity coefficient is calculated based on the *Smagorinsky* formula, which is given as follows (Mellor, 1998):

(4) $$\upsilon_{t}=\frac{1}{2}C_{t}\Delta l^{2}{\left[{\left(\frac{\partial u}{\partial x}\right)}^{2}+\frac{1}{2}{\left(\frac{\partial v}{\partial x}+\frac{\partial u}{\partial y}\right)}^{2}+{\frac{\partial v}{\partial y}}^{2}\right]}^{\frac{1}{2}}$$

where Δl = mesh spacing and *C_t_ =* model coefficient (with values of 0.25–1.0). The default value of 0.28 was adopted for Ct in this study following the recommendation of the model developer. 

### 2.2. Simulation Area and Scenarios

#### 2.2.1. Simulation Area

**Figure 1:**
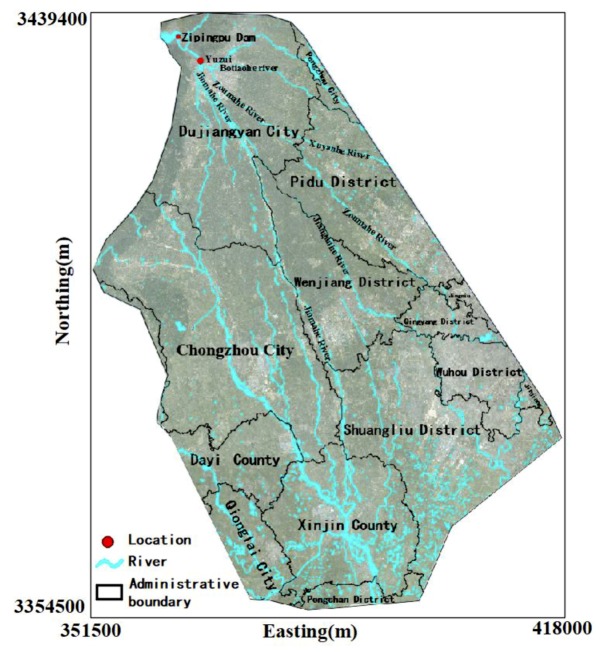
Simulation area.

The dam-break flood inundation analysis area mainly stretches from Dujiangyan City to Xinjin County downstream of the Zipingpu Reservoir, including Dujiangyan City, Chongzhou City, Xinjing County, Wenjiang District, Pidu District, and the main urban areas of Chengdu Qingyang district and Wuhou district (Figure 1). The computational domain is derived from the terrain file provided by the local bureau of surveying and mapping. The total area is 3160 km^2^. 

#### 2.2.2. Simulation Scenarios

The Zipingpu Reservoir dam is a concrete-faced rockfill dam with a maximum height of 156 m, a length of 663.77 m, and a crest width of 7.4 m. The crest elevation is 884 m, and the wave wall top elevation is 885 m. The construction site elevation is 728 m. This paper examines the emergency management of Zipingpu. Because the Zipingpu dam is a faced rockfill dam, it is more likely to collapse gradually than quickly. Therefore, the gradual collapse condition was selected in this scenario. In this paper, the Dambreak program is used to calculate the flow duration curve of a dam breach under the gradual collapse scenario (with an outburst time of 3 h), different water levels (the normal water level of 877 m and the check flood level of 883.1 m), different dam break breach elevations (set according to the elevation of the berms), and different bottom widths of the dam break breach and slopes of the breach. Four scenarios are chosen to simulate the evolution of a dam-break flood according to the maximum possibility, which is the most unfavorable representative perspective, using the engineering characteristics of the Zipingpu concrete-faced rockfill dam presented in Table 1. The representative meaning of scenario 1 and scenario 2 is that when a failure of the reservoir engineering facilities or excessive flooding occurs, the water level in front of the dam will rise, and the dam will break. Considering that the dam is at a normal water level for a relatively large amount of the year, if the dam break is caused by an earthquake, there is a high probability that the water level in front of the dam will be at a normal water level. Therefore, from the unfavorable perspective, two scenarios of outbursts under a normal water level are selected as scenario 3 and scenario 4, respectively, in this study. Scenarios 3 and 4 can also represent the dam failure scenarios caused by wars or terrorist attacks.

**Figure 2:**
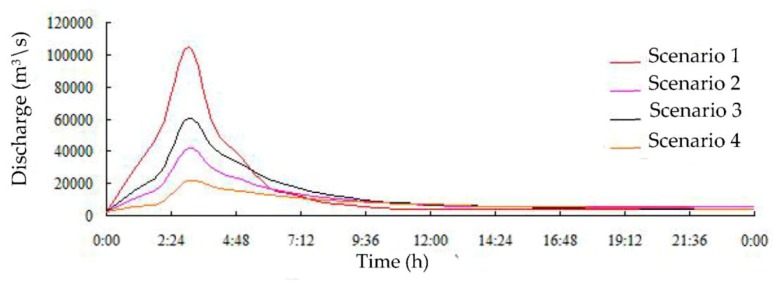
Dam site flow duration curves for each scenario.

The edge of the dam axis of the Zipingpu Reservoir is used as the boundary of the inlet. The discharge process of the dam break breach is used as the calculation inlet condition. The flow duration curves of the four scenarios are shown in Figure 2. The calculation time of each scenario is 24 h. 

### 2.3. Analysis of the Water Balance in Each Scenario

The water balance is adopted to analyze the rationality of the simulation results of the model. Since the initial water volume in the simulation area is zero, the sum of the water volume in the simulation area and the water volume flowing out of the boundary should be consistent with the water volume flowing into the inlet. The water quantity statistics of each scenario are shown in Table 2. Through statistical analysis of the water amount, it can be seen that the maximum error of the water amount into and out of each scenario is only 1.6%, and the water amounts flowing in and out are basically balanced.

### 2.4. Analysis of the Simulation Results

According to the flow rate at the dam site, the flow rate reaches a peak during the third hour. Between 0 and 3 h, the flow rate increases rapidly. During this period, the flow rate in each scenario varies considerably, and the flow rate reaches a peak from smaller values. After 3 h, the flow begins to decrease gradually. The flow rate decreases rapidly between 3 and 6 h. After 6 h, the flow rate changes only slightly. After 8 h, the flow rate value remains unchanged.

**Figure 3:**
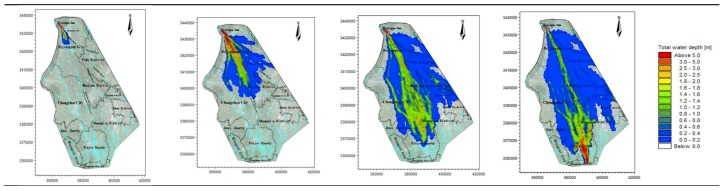
Scenario 1: Inundation at four time points.

**Figure 4:**
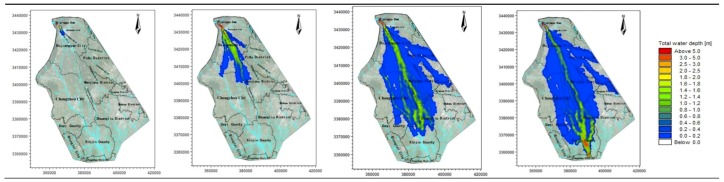
Scenario 2: Inundation at four time points.

**Figure 5:**
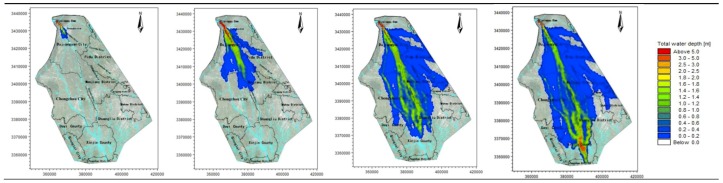
Scenario 3: Inundation at four time points.

**Figure 6:**
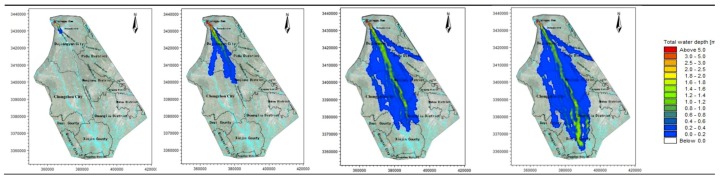
Scenario 4: Inundation at four time points.

**Figure 7:**
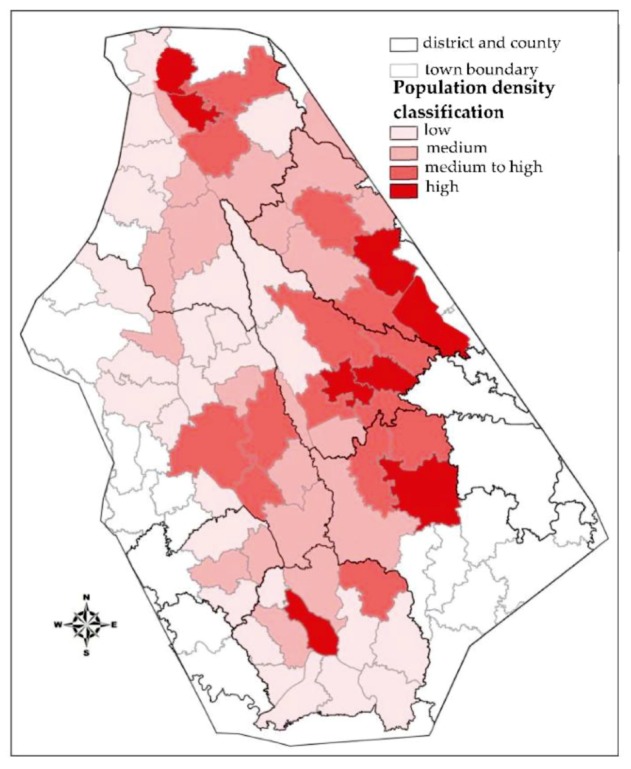
Population density of each unit.

Therefore, according to the time spots characteristic of the hydrograph, the four time spots of 1, 3, 6, and 8 h are used to analyze the flooding situation of the entire simulation area. The average water depth and velocity at all moments are shown in Table 3, and the water depth simulation results of the simulation area are shown in Figure 3, Figure 4, Figure 5, Figure 6 and Figure 7.

The water depth distribution map illustrates the following:

1. In the beginning of the evolution, both sides of the river are mountains, the flood is constrained within the channel of the river, and the water is deeper. After the flood reaches Yuzui, it enters the Chengdu plain, where the topographic constraints of the flood are reduced, and the flooding range increases rapidly. The average water depth of the entire flooding area is 0.76–1.72 m.
2. According to the water depth map, the Zipingpu dam-break flood mainly occurs along the river network in the calculation area, and the majority of the water flows along the Jinma River and the Western river. When the water is deeper (scenarios 1 and 3), the Jinma River and Western river are unable to channel the flood, and part of the river water flows along the Jianganhe River, Puyanghe River, Baitiaohe River, and Xuyanhe River. The flooding in scenarios 2 and 4 is relatively low, and the floods will not flow to the east.
3. When the flood reaches the flooding area, the flood will not be confined to the river because of the large area of the flood. Therefore, the water level does not change considerably due to the change in the flow at the dam site. The different flooding areas are mainly caused by changes of the entrance flow in each scenario.
4. By comparing the water depth map of each scenario, we can deduce that the larger the discharge, the faster the flood velocity will be and the deeper the water depth will be.
5. The flood striker enters Pengshan County after 7.4 h. After long-distance transmission, the flood process has become gentle, the flood peak decreases, and a considerable amount of water is stranded in the flooding area and flows to other rivers. The flood pressure of the downstream Jinmahe River decreases considerably, and the threat of flooding to the downstream area gradually decreases. The topography on both sides of the southern river is higher, which restricts the horizontal expansion of the flood. As a result, the flood will not threaten the downstream areas.
6. Under a large dam flood (scenario 1), the Qingyang, Jinniu, and Wuhou districts of Chengdu are affected, but the overall impact is not significant, as the maximum depth is only 0.61 m at the Wan Shouqiao position of the Wuhou district and the maximum flow rate is only 0.8 m/s. Scenarios 2–4 show no significant effects.

## 3. Method for Classifying the Flood Areas

### 3.1. Establishment of the Analysis Unit 

To cope with a flooding hazard, one should first establish the object to be protected under the flood risk and then classify the hazard factors of the object and the vulnerability of the environment, including the regional influence degree and regional population, to reduce the loss caused by a flood disaster. From the perspective of urban planning management, this paper presents a proposed method for classifying protection objects under flood risks with multiple factors. The township-, town-, and subdistrict-level administrative regions are designated as the units of analysis, and the flood protection objects are classified based on the analysis unit. According to the analysis results of the flood evolution presented in the previous chapter, the objects to be protected in the case of failure of the Zipingpu Reservoir are mainly 10 downstream districts (city) and counties under the jurisdiction of Chengdu City. The Qingyang District, Jinniu District, and Wuhou District of Chengdu City are affected by the flood flow rate (scenario 1), but the overall effect is not significant. Moreover, in scenarios 2–4, these areas are only slightly affected; thus, the Qingyang District, Jinniu District, and Wuhou District are not included in the analysis unit. 

### 3.2. Importance Classification of the Analysis Unit 

The dam flood impact area is wide, and the development of each region is different. The allocation of resources would not be reasonable if each region were to perform the same protection countermeasures. Instead, the resources should be allocated according to the partitioning importance level of each protection area. The index system allows for comprehensive consideration based on the risk concept framework of the objective, systematic, scientific, and operability principles to protect the area of influence to ensure the safety of people’s lives and property security, combined with the actual scenario of the study area. The chosen indices must be supported by sufficient data, as a very detailed damage assessment based on sparse data may be misleading [23]. The system is used to select values of the three indicators—population density, key protection objects, and industry output—to describe the regional importance level. Among these indicators, the key protection objects are locations where the flood affects a high-density area or locations where evacuation would be difficult, such as hospitals and schools. 

The risk simulation should involve the most unfavorable scenario to ensure the safety of people’s lives and property in the downstream area. Scenario 1, which is the dam-break flood with the most extensive impact and that causes the highest degree of disaster, is the most adverse scenario and is thus used as the flood risk management scenario. The basic information of the 71 analysis units for scenario 1 are shown in Supplementary Materials Table S1.

#### 3.2.1. Population Density 

The population distribution is an important factor impacting the loss caused by a flood disaster. If there is an area with no human economic activity and no population, then a flood will cause no loss and there will be no flood disaster, regardless of the magnitude of the flooding. Ensuring the safety of people is the primary task in mitigating flood disasters. The population density represents the density of people in an area. Therefore, the population density is one of the important indicators of the analysis unit. Referring to the management methods of relevant local government departments, this paper divides each unit into four levels according to the population density. First, the population density in each unit is sorted in descending order. The top 10% of units in this order are treated as high-level areas. Units 10%–30% are treated as middle-high-level areas. Units 30%–70% are treated as medium-level areas, and the bottom 30% of units are low-level areas. The population classification of each unit is shown in Figure 7. 

#### 3.2.2. Number of Hospitals and Schools in Flood-Affected Areas 

**Figure 8:**
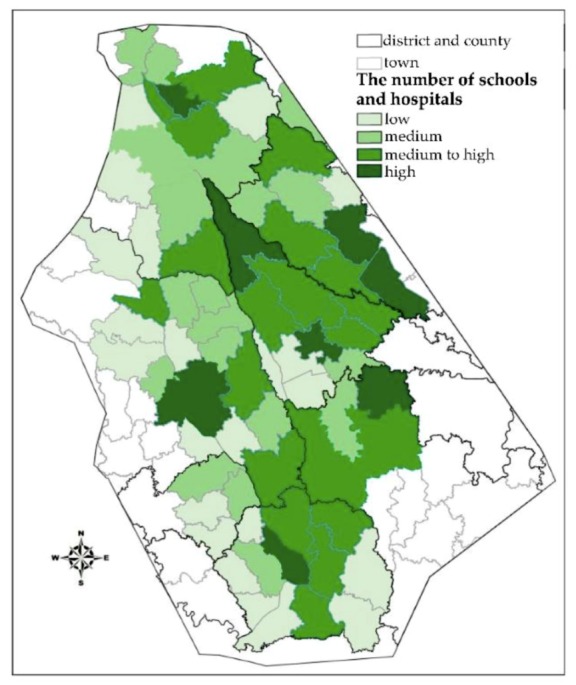
Number of hospitals and schools in each unit.

Hospitals and schools are places with high concentrations of people, which hinders the evacuation of people from such places. Moreover, secondary disasters, such as panic-stricken stampede accidents, can easily occur in such locations. In addition, inpatients and pupils are relatively weak in terms of both their self-protection and emergency escape capabilities; thus, such persons are categorized as “vulnerable” individuals in flood disasters. Hospitals and schools can also provide evacuation sites to rescue wounded individuals in disasters. Therefore, the number of hospitals and schools in flood-affected areas is also considered in the evaluation of regional importance. The number of hospitals and schools in each unit is shown in Figure 8. 

#### 3.2.3. Total Industrial Output 

**Figure 9:**
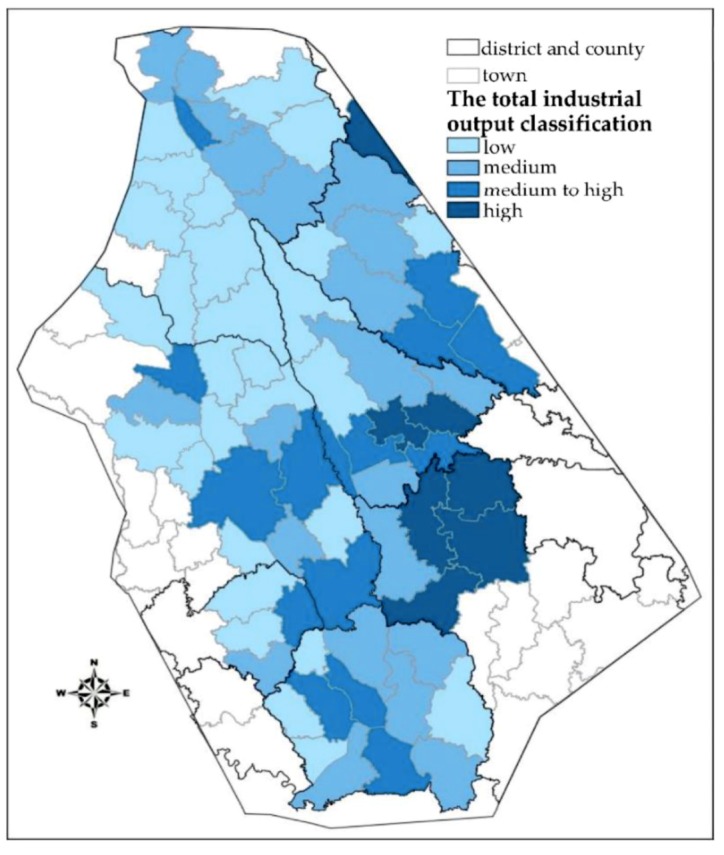
Total industrial output of each unit.

With the more developed economy and increased scale resulting from urban development, a city will suffer more losses in a flood disaster compared to the losses of rural areas. In the estimation of flood losses, the loss of industrial output accounts for a large proportion of the losses. Since the primary administrative units at the township level do not have statistics on the regional GDP, this paper uses the industrial output in each region to characterize the economic development of the region. The industrial output classification is shown in Figure 9. 

**Figure 10:**
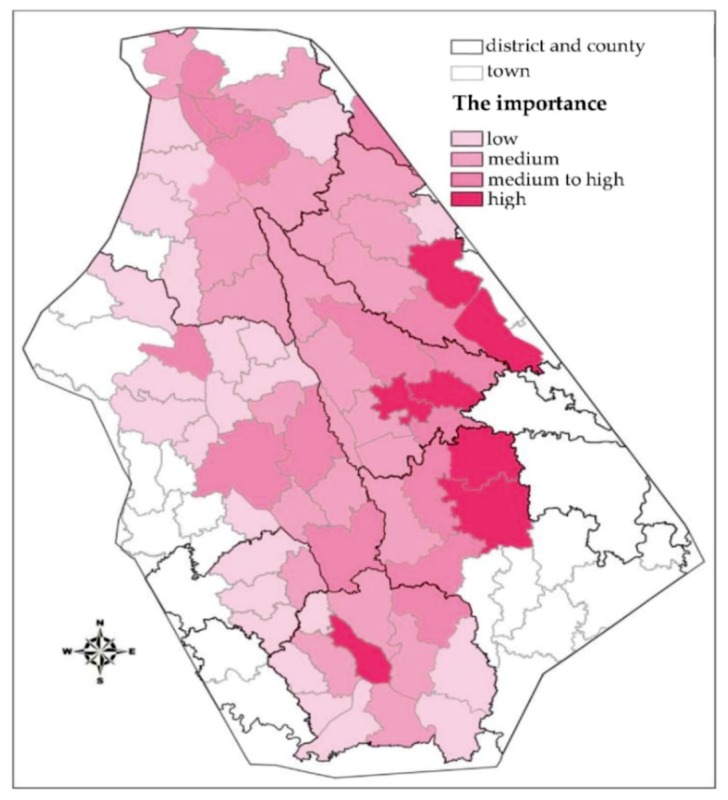
Importance level of each unit.

In recent years, the AHP has been usually used to rate the index factors of flood risks in flood risk assessments [24,25]. However, the data source of the AHP method is mainly expert grading, which is unavoidable due to interference from subjective factors. The three indicators selected in this paper are the most important factors in flood protection; therefore, the influences of the three indices on the importance of the unit are superimposed on each other. By superimposing the hierarchical images of the above three indices, the importance level of each unit can be confirmed. There are four levels under each index, and the superposition is too large to facilitate risk management and urban planning for a flood disaster. Therefore, the results of the three indices must be classified after superposition. Similarly, the importance level is divided into four grades according to the previous classification method, and the results are shown in Figure 10.

### 3.3. Flood Hazard Classification of the Analysis Unit 

Information on the flood risk factors can be obtained from hydraulics, hydrology, and the calculation of the flood impact estimation model. This information reflects the details of each flood risk factor and the spatial distribution characteristics, such as the submerged range, submerged water depth, flood velocity, arrival time, and duration of being submerged.

According to the previous scenario, the importance classification of flood-affected areas is mainly based on people’s lives and property in the region. The time duration before flood arrival determines the time that people have to perform evacuations and transfer property. The loss of property and lives in a unit area will be higher when the amount of time before flood arrival is shorter. Moreover, the submerged range, submerged water depth, and flood velocity can represent the disruptive strength in the analysis unit [26,27]. Based on the analysis of the simulation results in scenario 2, the flood flow velocity is higher when the submerged depth of the dam break flood is greater. Thus, the flood velocity is not treated as a flood hazard index. Instead, the flood arrival time, flood submerge range, and water depth are considered as the grading indices of dam-break flood risks. By summarizing historical data, Jonkman et al. determined that areas where the flood speed is over 0.5 m/h are rapid-growth areas of a flood [28], and the rapidly increasing flood speed in these areas prevents the population under risk from evacuating in time [29]. The analysis results in scenario 2 demonstrated that the flow of a dam break flood reached the peak in 0 h to 3 h, and the submerged water depth of the corresponding region also increased rapidly. Therefore, an area with a water depth of more than 0.5 m can be defined as a rapid-growth area of a flood. Also, the NR&M indicates that inundation depths lower than 0.5 m provide no imminent danger [30]. Thus, a flood with a submerged water depth of over 0.5 m is identified as a threat to lives and property. To consider the unit risk scenario under the effect of a dam break flood directly, the water depth and submerged range are combined into the submerged range with a water depth over 0.5 m to present the disruptive ability of a dam-break flood.

Because the area of each unit is different, the submerged areas cannot be compared horizontally; thus, the flooded area percentage is used to estimate the damage level. Therefore, this percentage is used in the classification of the flood risk index. The flood arrival time and percentage of submerged areas at over 0.5 m in each unit are shown in Supplementary Materials Table S2. 

The flood risk level cannot be divided according to the quantile of unit numerical order. Instead, it should be divided according to the actual meaning of the index. Historical data and practical experience demonstrate that it is difficult for people to evacuate and transfer property when the flood arrives within less than 3 h, and the disaster situation in a region is quite serious when the inundation ratio is greater than 40%. Accordingly, this paper divides flood risks into three levels:

1. Time and inundation high-risk areas: The flood arrival time is less than 3 h, and the flooding ratio is greater than 40%.
2. Time high-risk areas: The flood arrival time is less than 3 h, and the inundation ratio is less than 40%.
3. Inundation high-risk areas: The inundation ratio is greater than 40%, and the flood arrival time is greater than 3 h.

**Figure 11:**
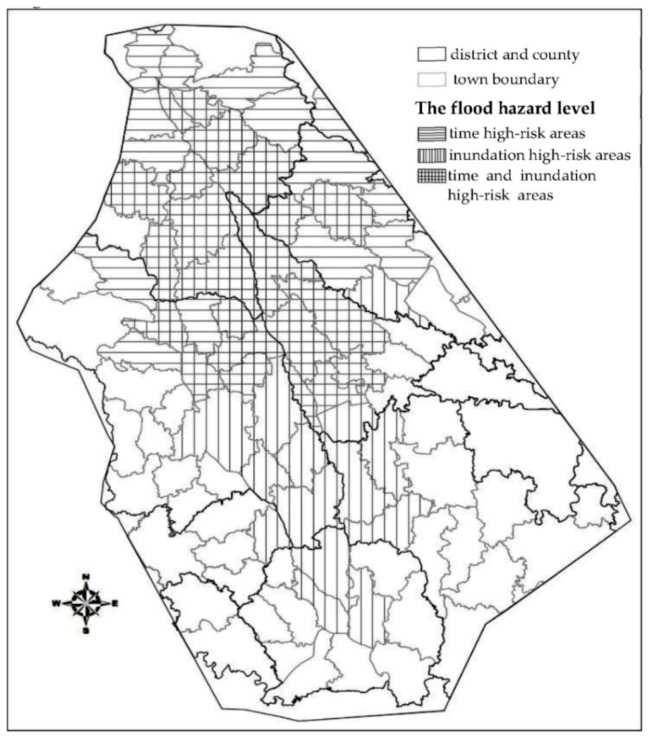
Dam-flood hazard map.

According to this classification method, the flood risk level of each unit is shown in Figure 11.

### 3.4. Dam-Flood Risk Map Incorporating Importance and Flood Hazard Levels

**Figure 12:**
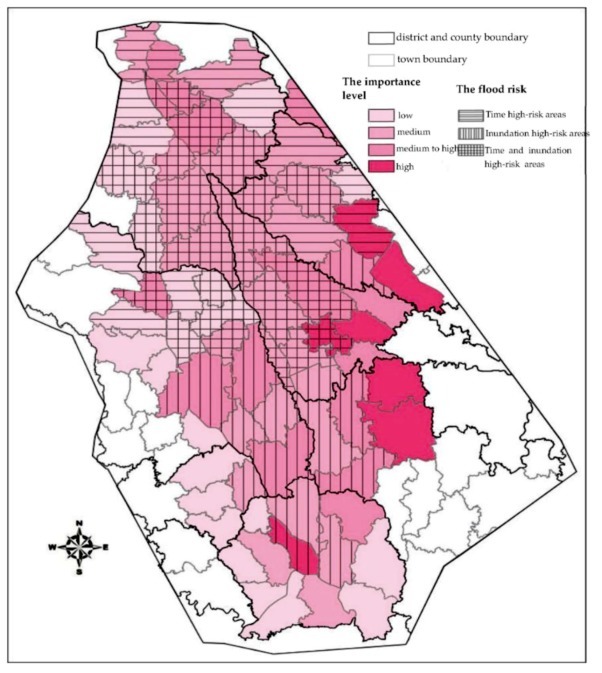
Flood risk map incorporating importance and flood hazard levels.

A dam-break flood risk map based on multiple criteria can be obtained by superimposing the importance grading map with the risk grading map (Figure 12).

## 4. Management Countermeasures for Flood-Affected Areas 

Based on previous experience in flood risk management, the countermeasures used in flood risk management can be summarized into the following three aspects:

1. Regulatory countermeasures. Through rational planning, scientific management and optimal dispatching of flood control and waterlogging control engineering systems, and comprehensive utilization of prevention, drainage, storage, stagnation, seepage, and other means can effectively reduce risks.
2. Adaptive countermeasures. Through the scientific formulation of urban development and land use planning, measures such as building flood resistance protection and flood insurance can be taken to enhance the ability to adapt to risks.
3. Emergency countermeasures. Through the construction of urban emergency response and coordination linkage systems, scientific formulation of pre-warning plans with preparations and exercises in advance, timely forecasting, early warning, effective organization of emergency rescue, quick repair of flood-damaged facilities, restoration of normal order, etc., can enhance the ability to bear risks.

According to the integrated dam-break flood risk map in the previous chapter, there are 12 types of units, as shown in Table 4.

Units considered time high-risk areas are mainly distributed in Wenjiang District and north of Wenjiang District. When developing flood management countermeasures, these units should focus more on emergency countermeasures. Among these units, 11 are medium or low in importance. Most of these units are located in rural areas with weak infrastructure and no good escape conditions, which should be paid special attention when developing countermeasures.

Inundation high-risk areas are mainly distributed along the Jinma River, and these units should give more attention to adaptive countermeasures. There is one high-importance unit and six medium-high-importance units among these units; thus, more flood control facilities should be built, and soil and water conservation should be strengthened.

Units of high risk and importance level should not only develop good adaptive countermeasures and emergency countermeasures but should also focus on regulatory countermeasures. According to the importance level of each unit, the flood control resources should be allocated rationally, and the flood control countermeasures should be formulated more pertinently.

For other units, supervision and control should also be established to avoid secondary injury.

## 5. Conclusions

Due to the increased urbanization of Chengdu in recent years, integrated flood management has become increasingly important. This study analyzed the flood risk due to a dam break in the Zipingpu Reservoir. The study focused mainly on an assessment of the risk of a dam-break flood in the Zipingpu Reservoir to Chengdu City.

From the perspective of urban management, we proposed a type of dam-break flood risk management method that uses administrative divisions as analysis units to bridge the gaps of previous research. By using the township-, town-, and subdistrict-level administrative regions as the analysis units, the protection objects of flood risk management were made more explicit, and the administrative resources allocation was more detailed. The method of classifying the analysis units is based on the results of a numerical simulation and a large amount of geographical, social, economic, and population data, and this method makes urban flood risk management more refined and effective. 

**Highlights**

- Subdistrict-level administrative regions are designated as the units of analysis for flood risk management.
- A classification method for flood hazard and unit importance is proposed.
- An integrated flood risk map is drawn for the flood risk management of Zipingpu Reservoir.

## Figures and Tables

**Table 1:** Dam break parameters of each scenario.

Scenario	Water Level (m)	Dam Break Time (h)	Dam Break Parameters			Maximum Discharge During a Breach (m^3^/s)	Explanation
			Dam Break Breach Elevation (m)	Bottom Width of Dam Break Breach (m)	Slope of Breach		
1	883.1	3	796	100	0.5	105,544	Gradual collapse considering the slope elevation
2	883.1	3	840	100	0.5	41,658	Failure of the dam structure
3	877	3	804	50	0.5	60,219	Earthquake, war, or terrorist attack
4	877	3	840	50	0.5	21,408	Non-break flood or dead water level bursts

**Table 2:** Statistical table of the simulation area water inflow and outflow.

Scenario	The Water Volume Flowing in the Inlet (m^3^)	Simulation Area Water Statistics (m^3^)				Error Rate (%)
		The Water Volume Flowing Out of Chengdu, Shaungliu, Pidu	The Water Volume Flowing Out of Xinjing	The Amount of Water Stuck in the Simulation Area	Total	
1	13.77 × 10^9^	0.70 × 10^9^	10.04 × 10^9^	3.26 × 10^9^	14.00 × 10^9^	1.6
2	9.41 × 10^9^	0.003 × 10^9^	6.135 × 10^9^	3.20 × 10^9^	9.34 × 10^9^	0.7
3	11.53 × 10^9^	0.096 × 10^9^	8.244 × 10^9^	3.20 × 10^9^	11.54 × 10^9^	0.7
4	6.97 × 10^9^	0	4.37 × 10^9^	2.70 × 10^9^	7.07 × 10^9^	1.5

**Table 3:** Statistical table of the simulation area water inflow and outflow.

**Time**	**Scenario 1**				**Scenario 2**			
	**Depth at Yuzui (m)**	**Velocity at Yuzui (m/s)**	**Flooding Area Average Depth (m)**	**Flooding Area Average Velocity (m/s)**	**Depth at Yuzui (m)**	**Velocity at Yuzui (m/s)**	**Flooding Area Average Depth (m)**	**Flooding Area Average Velocity (m/s)**
1 h	2.13	3.08	2.82	3.73	1.06	1.98	2.79	3.67
3 h	7.22	6.90	1.72	3.31	2.93	3.59	1.59	3.17
6 h	1.94	2.93	1.03	1.85	1.62	2.64	0.87	1.69
8 h	1.13	2.06	1.08	1.56	1.29	2.26	0.82	1.57
**Time**	**Scenario 3**				**Scenario 4**			
	**Depth at Yuzui (m)**	**Velocity at Yuzui (m/s)**	**Flooding Area Average Depth (m)**	**Flooding Area Average Velocity (m/s)**	**Depth at Yuzui (m)**	**Velocity at Yuzui (m/s)**	**Flooding Area Average Depth (m)**	**Flooding Area Average Velocity (m/s)**
1 h	1.34	2.33	2.72	3.80	0.68	1.30	2.86	2.82
3 h	4.36	6.18	1.65	3.22	2.02	2.99	1.41	2.81
6 h	2.10	3.05	0.97	1.81	1.39	2.39	0.77	1.66
8 h	1.46	2.46	0.92	1.64	1.20	2.15	0.76	1.48

**Table 4:** Unit type statistics.

	The Flood Risks	The High-Risk	Inundation High-Risk	Time and Inundation High-Risk
The Importance Level				
High		1	1	1
Medium to high		2	6	4
Medium		4	7	10
Low		7	0	5

## Supplementary Materials

The following are available online at https://www.mdpi.com/1660-4601/16/19/3786/s1.
