# Supplementary material for: An Approach Based on the Protected Object for Dam-Break Flood Risk Management Exemplified at the Zipingpu Reservoir
Source: Int J Environ Res Public Health. 2019 Oct 8;16(19):3786. doi: 10.3390/ijerph16193786 (PMC6801503; doi:10.3390/ijerph16193786)
Supplement: Supplementary file 1 [file ijerph-16-03786-s001.pdf]

International Journal of
Environmental Research and Public Health

MDPI

Supplementary materials

Table 1. The basic information of the 71 analysis units.

Analysis unit	Population density (people/ square kilometer)	Number of hospitals and schools	Total industry output (ten thousand yuan)
Zipingpu	195.20	3	80,981.00
Guankou	3,994.37	3	12,793.50
Xingfu	4,726.35	9	113,097.20
Binjiang	1,121.14	4	153,254.40
Yutang	455.56	1	6,075.00
Xujia	1,166.71	5	23,792.70
Zhongxin	615.25	3	9,862.00
Juyuan	1,293.31	5	42,500.00
Tianma	882.60	1	30,763.00
Qingchenshan	343.03	2	14,846.00
Cuiyuehu	915.31	3	390.00
Chongyi	923.80	3	73,501.00
Shiyang	924.90	3	7,760.00
Liujie	827.17	6	18,000.00
Anlong	903.71	2	24,966.00
Lichun	894.30	3	363,257.00
Shouan	821.94	9	38,216.00
Hesheng	839.19	6	23,675.00
Wanchun	1,366.29	5	42,727.00
Tianfu	2,666.67	2	301,254.00
Liucheng	13,600.00	13	403,215.00
Gongping	4,333.17	4	356,541.00
Yongquan	3,100.78	3	286,521.00
Yongning	1,245.42	4	110,544.00
Jinama	1,004.81	2	56,101.00
Yongsheng	1,002.00	2	156,000.00
Tangchang	969.90	8	57,567.00
Huayuan	1,073.72	3	15,191.00
Ande	1,210.13	3	78,420.00
Xinminchang	986.24	2	19,018.00
Youai	1,019.98	4	53,918.00
Pitong	6,269.59	12	201,214.00
Deyuan	1,703.65	4	198,546.10
Hezuo	4,484.30	10	185,421.00
Jiezi	817.19	1	15,675.00
Yuantong	985.05	4	120,187.00
Guansheng	861.10	3	29,469.00
Zitong	883.77	3	30,700.00
Gongyi	518.42	2	66,318.00
Jinjiang	825.54	2	36,220.00
Liaojia	856.22	3	8,247.00
Chongpin	964.60	3	105,160.00
Daoming	553.66	0	18,757.00

Chongyang	1,712.78	16	222,442.00
Jixie	874.60	3	26,727.00
Yangma	1,236.69	7	148,310.00
Jixina	814.55	2	16,235.00
Dahua	1,206.22	2	53,060.00
Jiangyuan	1,138.18	3	6,330.00
Sanjiang	1,155.14	6	118,056.00
Dongchang	777.43	2	13,845.00
Caichang	930.65	3	5,375.00
Shaqu	934.42	3	224,551.00
Hanchang	853.82	2	64,273.00
Jinqiao	1,053.93	4	40,200.00
Pengzhen	1,380.46	3	734,867.00
Jiujiang	2,702.70	12	654,123.00
Dongsheng	3,796.99	5	895,412.00
Huangshui	1,099.97	5	998,841.00
Wenjin	684.36	2	32,546.00
Xingyi	917.85	4	41,052.00
Huayuan	1,589.55	6	49,526.00
Fangxing	739.13	2	31,312.00
Xinping	973.99	3	146,186.00
Wujing	3,761.72	10	154,396.00
Huaqiao	887.36	4	70,546.00
Puxing	575.99	2	73,495.00
Anxi	878.31	2	31,742.00
Yongshang	805.55	2	70,689.00
Dengshaung	858.38	5	117,127.00
Jinhua	564.21	0	46,350.00

Table 2. flood arrival time and submerged area percentage of each unit.

Analysis unit	Flood arrival time(min)	Submerged area percentage
Zipingpu	5	10.70%
Guankou	30	0.36%
Xingfu	45	56.34%
Binjiang	30	99.66%
Yutang	35	20.17%
Xujia	120	2.16%
Zhongxin	55	33.93%
Juyuan	55	76.54%
Tianma	150	17.56%
Qingchenshan	80	3.23%
Cuiyuehu	60	93.49%
Chongyi	75	65.72%
Shiyang	80	74.87%
Liujie	100	76.89%
Anlong	95	63.31%
Lichun	180	4.95%
Shouan	85	75.86%
Hesheng	130	91.35%
Wanchun	135	42.57%
Tianfu	170	78.98%
Liucheng	175	47.96%
Gongping	205	12.57%
Yongquan	200	27.49%
Yongning	230	28.98%
Jinama	195	88.74%
Yongsheng	155	99.92%
Tangchang	110	19.21%
Huayuan	110	52.89%
Ande	120	40.28%
Xinminchang	150	23.48%
Youai	140	34.95%
Pitong	155	11.80%
Deyuan	185	60.65%
Hezuo	210	37.37%
Jiezi	120	10.73%
Yuantong	130	8.71%
Guansheng	155	20.82%
Zitong	125	99.81%
Gongyi	170	14.38%
Jinjiang	175	44.72%
Liaojia	130	88.21%
Chongpin	160	93.79%
Daoming	185	4.27%
Chongyang	185	47.30%
Jixie	195	20.70%
Yangma	155	100.00%
Jixina	240	39.06%
Dahua	205	91.24%
Jiangyuan	200	98.75%
Sanjiang	225	98.75%
Dongchang	255	8.93%
Caichang	315	4.50%

Shaqu	260	44.24%
Hanchang	350	5.27%
Jinqiao	205	97.92%
Pengzhen	215	68.76%
Jiujiang	260	5.59%
Dongsheng	245	8.16%
Huangshui	265	85.61%
Wenjin	280	35.19%
Xingyi	275	93.08%
Huayuan	285	78.20%
Fangxing	375	8.67%
Xinping	335	36.75%
Wujing	300	96.32%
Huaqiao	300	95.30%
Puxing	340	14.09%
Anxi	410	26.58%
Yongshang	385	10.38%
Dengshaung	360	48.90%
Jinhua	385	18.24%

© 2019 by the authors. Submitted for possible open access publication under the terms and conditions of the Creative Commons Attribution (CC BY) license (http://creativecommons.org/licenses/by/4.0/).
